# Transparent and accurate reporting increases reliability, utility, and impact of your research: reporting guidelines and the EQUATOR Network

**DOI:** 10.1186/1741-7015-8-24

**Published:** 2010-04-26

**Authors:** Iveta Simera, David Moher, Allison Hirst, John Hoey, Kenneth F Schulz, Douglas G Altman

**Affiliations:** 1 Centre for Statistics in Medicine, University of Oxford, Oxford, UK; 2 Clinical Epidemiology Program, Ottawa Hospital Research Institute, Ottawa, Canada; 3 Queen's University, Kingston, Canada; 4 Family Health International, Durham; UNC School of Medicine, Chapel Hill, North Carolina, USA

## Abstract

Although current electronic methods of scientific publishing offer increased opportunities for publishing all research studies and describing them in sufficient detail, health research literature still suffers from many shortcomings. These shortcomings seriously undermine the value and utility of the literature and waste scarce resources invested in the research. In recent years there have been several positive steps aimed at improving this situation, such as a strengthening of journals' policies on research publication and the wide requirement to register clinical trials.

The EQUATOR (Enhancing the QUAlity and Transparency Of health Research) Network is an international initiative set up to advance high quality reporting of health research studies; it promotes good reporting practices including the wider implementation of reporting guidelines. EQUATOR provides free online resources http://www.equator-network.org supported by education and training activities and assists in the development of robust reporting guidelines. This paper outlines EQUATOR's goals and activities and offers suggestions for organizations and individuals involved in health research on how to strengthen research reporting.

## Introduction

Scholarly publishing is undoubtedly undergoing a revolution that has brought not only new ways of disseminating research information but also a more critical perspective on assessing published information. Some of these changes are driven by the new technologies; others have occurred as a response to the shortcomings of scholarly publishing such as difficulty of accessing published research or obtaining sufficient information from the published text. Journals remain the core of research communication but the way that this is now done has been transformed. The vast majority of scientific journals, including those publishing health research papers, are now available online [1]. This allows the supplementing of research articles with additional information or data, showing pre-publication history or readers' comments on the published research. Some of these features have been facilitated by open access publishing, which has grown from a brave experiment into a highly regarded and frequently used publication method. In recent years we have also seen a growing awareness of ethical issues and an acknowledgement of widespread problems influencing the reliability of the published medical research literature [2-5]. Such problems also extend to the process of manuscripts' peer review [6].

Some journals have taken steps to improve the quality of the research they publish. These include improving their instructions to authors [7-9], introducing new thematic sections for articles on research methodology and guidance [10,11] and a requirement to register clinical trials [12] or deposit data into public repositories [7,13].

All these new trends have the potential to contribute to an increased transparency in health research publishing. However, despite these initiatives many problems in the research literature still prevail. In this article we will focus on the inadequate quality of reporting of health research and highlight current initiatives to improve the situation.

## How reliable are published reports of health research?

A growing body of methodological research reviewing the published literature highlights a variety of shortcomings in health research publications. Appendix 1 lists some of those problems. Such deficiencies make it difficult, or impossible, to assess how the research was conducted, to evaluate the reliability of the presented findings or to place them in the context of existing research evidence. As a result published studies often cannot be used by clinicians in patients' care [14] or to inform public health policy [15]. The preferential publication of positive results leads to an overestimation of the benefits of new therapies, which may lead to increased costs without a corresponding improvement in patient outcomes [5]. Non-publication, or biased reporting of research findings, may indirectly harm patients including those involved in future research [5]. Particularly problematic and unethical is the insufficient or misleading reporting of adverse events (harms) in randomized trials [16].

## What is being done to improve the reporting of health research?

Some of these problems can be addressed by the wider and more consistent use of reporting guidelines. Reporting guidelines provide advice on how to report research methods and findings. Usually in the form of a checklist, a flow diagram or explicit text, they specify a minimum set of items required for a clear and transparent account of what was done and what was found in a research study - reflecting, in particular, issues that might introduce bias into the research. Most robust and widely recognized guidelines have been developed systematically; they incorporate relevant available evidence and reflect consensus opinion of experts in a particular field, including research methodologists and journal editors [17]. Reporting guidelines complement general advice on the basic principles of scientific writing and specific journals' instructions to authors [18,19].

A large number of reporting guidelines have been published during the last 15 years [20]. As there is no consensus on how these guidelines should be developed, they represent a rather heterogeneous group in terms of their scope, development methodology and presentation of recommendations [17]. Some of these guidelines (see Table 1) are already widely endorsed by journals and, thus, are intended to be followed by their contributing authors. Although initial studies evaluating the impact of journal support for reporting guidelines indicate beneficial effects on the completeness and transparency of publications [21,22], there is still room for better implementation of and adherence to these guidelines [23,24].

**Table 1:** Reporting guidelines for the main study types. More than 90 reporting guidelines are included in the EQUATOR Network's online Library for Health Research Reporting at http://www.equator-network.org/.

CONSORT	Randomized trials	http://www.consort-statement.org/
STROBE	Observational studies	http://www.strobe-statement.org/

STARD	Diagnostic accuracy studies	http://www.stard-statement.org/

PRISMA	Systematic reviews and meta-analyses	http://www.prisma-statement.org/

SQUIRE	Quality improvement studies	http://www.squire-statement.org/

There is growing evidence that use of a checklist - a core part of a reporting guideline - is a beneficial tool. For example, within surgery the use of a checklist was associated with saving lives and reducing morbidity [25].

## The EQUATOR Network: helping to improve research reports

The EQUATOR Network (Enhancing the QUAlity and Transparency Of health Research) is an international initiative set up to help improve the reliability and value of health literature by promoting responsible reporting of health research [4,26].

Appendix 2 summarizes the major goals of the EQUATOR Network. The main focus is on dissemination of the basic principles of responsible research reporting and the wider implementation of reporting guidelines. The EQUATOR website provides an up to date centralized resource for: researchers writing up their studies (notably guidance on reporting, scientific writing, ethical conduct in research and publication); for peer reviewers assessing research manuscripts; for editors who wish to implement policies to aid accurate and transparent research reporting in their journals; and also for scientists wishing to develop further high quality reporting guidelines http://www.equator-network.org/. Recently, we published an overview of our online resources and a summary of available guidelines to allow better dissemination of this information [20]. Every 3 months we issue an online newsletter highlighting new reporting guidelines and other information relevant to responsible reporting. Our website statistics indicate a steadily growing global interest in these resources.

EQUATOR workshops developed by our team support the use of resources available on our website. They focus on journal editors as 'quality gatekeepers' but also on young researchers and research students in order to introduce good research reporting habits early in their scientific careers [27]. The work of journal editors and peer reviewers is difficult and an improvement in the quality of submitted manuscripts would ease the peer review process and reduce the number of problems that currently elude editorial and peer review checks [6].

The EQUATOR programme is very young and there are important areas of work that we still need to tackle. These include:

• Strengthening the methodology for the development and assessment of reporting guidelines

• Investigating the barriers and facilitators of reporting guideline use

• Increasing the awareness of the EQUATOR Network and the available resources worldwide and supporting the implementation of activities leading to better reporting of health research

It would be valuable to have generic reporting guidelines for all the main study types. These could then be implemented in specific areas of health research methodology and, if necessary, extended to, for example, address issues specific to clinical specialties. Harmonizing terminology, definitions of outcome measures and adverse effects seems to be next logical step to allowing better comparison across studies [28-30]. Well developed reporting guidelines have the potential to improve the clarity, completeness and transparency of research publications. Such publications can help us to gain the maximum value from funded research and may save lives and reduce the burden of illness on patients and costs to the healthcare system. Clearly, the reporting guidelines themselves must be robustly developed and should observe the same good reporting principles when presenting recommendations to their potential users. Drawing on our experience in the development of many reporting guidelines, members of the EQUATOR team recently published 'Guidance for Developers of Health Research Reporting Guidelines' [18].

We plan also to develop a reporting guideline assessment tool, similar in principle to the AGREE Instrument for clinical practice guidelines [31]. Our systematic review [17] revealed important differences among the available guidelines. An evaluation of reporting guidelines in relation to identified criteria of importance would help journal editors to choose the most suitable guidelines for their journal and to request an appropriate level of compliance.

The EQUATOR team is investigating the factors that prevent or facilitate the use of reporting guidelines; this work will be very helpful in formulating a strategy for better dissemination and routine implementation of the guidelines in practice. Meanwhile, we offer some advice and practical tips on how to promote accurate and transparent reporting and the wider use of reporting guidelines. Appendix 3 provides a summary; more details are on the EQUATOR website.

The way that readers seek, access and read articles has also changed. Readers are more likely to search for a specific article rather than browse through journal issues. They read more articles per year but spend less time on individual articles [1]. Readers do not have the time to try to work out what happened in a study and they should not have to. Authors must recognize the importance of clarity, structured format, logical flow of information and the key elements that should be reported; they should make it easy for readers to find the important information in their article. Peer reviewers should also take account of these principles when reviewing a manuscript.

It is important to increase awareness of the current poor level of reporting in the health research literature. Poor reporting may generally reflect an author's insufficient knowledge of good reporting practices and the reader's needs rather than deliberate attempts to mislead the readership. Whatever the reasons, it needs to be widely acknowledged that poor reporting is unacceptable. In this regard, we hope to assist by achieving increased awareness of the EQUATOR programme and our accumulated resources worldwide.

## Concluding remarks

The STM overview of scientific and scholarly journal publishing [1] shows that, despite a huge change in the way journals are published, researchers' core motivations for publishing have remained largely unchanged, focussing on securing funding and furthering their careers. We appeal to researchers, peer reviewers, journal editors, research institutions and funding agencies to consider how, and by whom, their research articles will be used and to consider whether the final papers meet the needs of these potential users. Using reporting guidelines checklists in writing and peer reviewing will increase the completeness, clarity and transparency of research papers without restricting researchers' creativity.

The available guidelines can help to raise the standard of research reporting but they need to be supported by the researcher's prior knowledge of the principles of good research conduct and reporting, ethical issues related to research and publication and basics of clear scientific writing. Many journals already champion the cause for a better quality of research reporting; however, others need to follow this path or take a more progressive lead. We all need to recognize that endorsement does not automatically translate into adherence. In order to truly change the reporting culture, everyone involved in the process of research and its publication needs to participate. Journals have a major role to play but, in addition, academic and other research organizations, funders and regulatory bodies need to become much more proactive in order to ensure that the exchange of research information is accurate, complete and transparent.

## Competing interests

All authors are members of the EQUATOR Network: Drs Altman, Moher, Schulz and Hoey are members of the EQUATOR Steering Group; Dr Simera and Mrs Hirst are supported by the EQUATOR programme funding.

## Authors' contributions

All authors contributed to the development of the EQUATOR Network vision, goals and specific activities which are described in this article. Dr Simera prepared the first draft of the manuscript and all co-authors contributed to revised drafts and have approved the final version.

## Appendix 1

### Shortcomings in health research reporting

The following practices cause major concern:

• Non-reporting or delayed reporting of whole studies [5,32]

• Selective reporting of only some outcomes in relation to study findings [33,34]

• The omission of crucial information in the description of research methods and interventions [14,21,35,36]

• Omissions or misinterpretation of results in abstracts [37]

• Inadequate or distorted reporting of harms [16,28,29,38]

• Confusing or misleading presentations of results, data and graphs [39,40].

## Appendix 2

### Seven major goals of the EQUATOR Network

1. Develop and maintain a comprehensive internet-based resource centre providing up-to-date information, tools and other materials related to health research reporting

2. Assist in the development, dissemination and implementation of robust reporting guidelines

3. Actively promote the use of reporting guidelines and good research reporting practices through an education and training programme

4. Conduct regular assessments of how journals implement and use reporting guidelines

5. Conduct regular audits of the reporting quality across the whole spectrum of health research literature

6. Set up a global network of local EQUATOR collaborating centres in order to facilitate the improvement of health research reporting on a worldwide scale

7. Develop a general strategy for translating the principles of responsible research reporting into practice.

## Appendix 3

### Steps to consider in order to support and practice accurate and transparent reporting of health research studies and promote the available resources

#### Journals

• Incorporate an explicit philosophy of transparent, complete and accurate reporting and the use of reporting guidelines into your organizational strategy/editorial policy

• Explore the available reporting guidelines; select well-developed guidelines appropriate for the reporting of research studies published in your journal

• Ask or instruct authors to adhere to these guidelines and motivate their use

• Ask or instruct peer reviewers to use the appropriate reporting guidelines when assessing manuscripts

• Refer to the EQUATOR Network website in your 'Instructions to Authors'.

#### Editorial organizations

• Inform your members of the existence of EQUATOR and its compiled online resources

• Refer to the EQUATOR Network and its resources on your website.

#### Research funding organizations

• Require accurate, complete and transparent reporting of the projects that you fund

• Alert researchers to available reporting guidelines and motivate their use

• Refer to the EQUATOR Network and its resources on your website.

#### Academic and other research institutions

• Promote and support accurate and transparent reporting of health research studies and the use of reporting guidelines through your policies, resources and educational activities

• Set aside resources to develop educational activities on reporting and peer reviewing research

• Consider the options of introducing education on good research reporting practice: incorporate in existing courses; include in 'research integrity' education; organize stand alone workshops developing reporting skills (online or face-to-face); organize seminars and talks highlighting the importance of accurate research reporting; include information about research reporting policies and EQUATOR resources in welcome pack for new research staff; and so on

• Refer to the EQUATOR Network and its resources on your website

• Display promotional materials available on the EQUATOR website introducing reporting guidelines and other resources (for example, in research departments; health research libraries, and so on).

#### Reporting guidelines developers

• Inform EQUATOR about your planned guideline development in order to avoid duplication and confusion.

#### Authors of research articles

• Find out about reporting requirements early when planning your research study

• When writing up your research, check the EQUATOR website for any new relevant guidelines in order to help improve the quality of your manuscript

• Adhere to the relevant reporting guideline(s); when not reporting on certain items explain the reason why. Remember that reporting guidelines provide a minimum set of items; other details specific to your particular study might be relevant for a clear and complete account of what was done and found (consider, in particular, items that might have introduced bias into your research). It is important to provide enough information to allow your study to be potentially reproducible by others.

## Pre-publication history

The pre-publication history for this paper can be accessed here:

http://www.biomedcentral.com/1741-7015/8/24/prepub
